# Biogeographic patterns of potential pathogenic bacteria in the middle and lower reaches of the Yangtze River as well as its two adjoining lakes, China

**DOI:** 10.3389/fmicb.2022.972243

**Published:** 2022-09-02

**Authors:** Xiaoling Wan, Jia Li, Shiyong Wang, Fei Fan, Richard William McLaughlin, Kexiong Wang, Ding Wang, Jinsong Zheng

**Affiliations:** ^1^The Key Laboratory of Aquatic Biodiversity and Conservation of the Chinese Academy of Sciences, Institute of Hydrobiology, Chinese Academy of Sciences, Wuhan, China; ^2^University of Chinese Academy of Sciences, Beijing, China; ^3^Changjiang Survey, Planning, Design and Research Co., Ltd., Wuhan, China; ^4^Key Laboratory of Changjiang Regulation and Protection of Ministry of Water Resources, Wuhan, China; ^5^General Studies, Gateway Technical College, Kenosha, WI, United States

**Keywords:** bacterial pathogen, distribution pattern, shaping factor, Yangtze River, high-throughput sequencing

## Abstract

Understanding the distribution patterns and shaping factors of bacterial pathogens in aquatic ecosystems, especially in natural waters, are critical to the control of pathogen transmission. In this study, using *16S rRNA* gene amplicon sequencing, we explored the composition and biogeographic dynamics of potential bacterial pathogens in the middle and lower reaches of the Yangtze River, as well as its two vast adjoining lakes (Dongting Lake and Poyang Lake). The pathogen community belonged to 12 potential pathogenic groups, with “intracellular parasites,” “animal parasites or symbionts” and “human pathogens all” occupying 97.5% in total. The potential pathogen community covered seven phyla with Proteobacteria (69.8%) and Bacteroidetes (13.5%) the most predominant. In addition, 53 genera were identified with *Legionella* (15.2%) and *Roseomonas* (14.2%) the most dominant. The average relative abundance, alpha diversity and microbial composition of the potential bacterial pathogens exhibited significant biogeographical variations among the different sections. An in-depth analysis reflected that environmental variables significantly structured the potential bacterial pathogens, including water physiochemical properties (i.e., chlorophyll-*a*, total nitrogen and transparency), heavy metals (i.e., As and Ni), climate (i.e., air temperature) and land use type (i.e., waters). Compared to the overall bacterial community which was composed of both pathogenic and non-pathogenic bacteria, the pathogen community exhibited distinct microbial diversity patterns and shaping factors. This signifies the importance of different variables for shaping the pathogen community. This study represents one attempt to explore pathogen diversity patterns and their underlying drivers in the Yangtze River, which provides a foundation for the management of pathogenic bacteria.

## Introduction

Various pathogenic agents have been detected in freshwater which provides drinking water and agricultural activities. These pathogens included bacteria (e.g., *Escherichia coli* O157:H7), fungi (e.g., *Aspergillus fumigatus*), enteric viruses (e.g., *Enterovirus*) and several protozoans (e.g., *Cryptosporidium*) (Ferguson et al., 2003; Hageskal et al., 2009). Among these, bacteria are the most frequently studied group owing to their wide varieties and relatively acute symptoms. For example, the water-borne epidemic of *Escherichia coli* O157:H7 can cause severe bloody diarrhea, leading to life-threatening haemolytic uraemia syndrome (Sharma et al., 2003). Moreover, infectious diseases arising from bacterial pathogens may also cause abnormal shoot proliferations of plants (Dhaouadi et al., 2020) and lead to reproductive failures in female animals, which may threaten their population growth (Langwig et al., 2015; Kamath et al., 2016). Recently, wastewater treatment technologies have achieved progress in eliminating pathogenic bacteria loads, however, waterborne infectious diseases due to bacterial pathogens, including newly emerging and re-emerging pathogens, are still globally a public health issue (Cissé, 2019; Motlagh and Yang, 2019; Jenkins et al., 2021). Common examples include *Helicobacter pylori* (Boehnke et al., 2018), *Escherichia coli* (Park et al., 2018) and *Campylobacter* outbreaks (Gilpin et al., 2020). Rivers can provide food and water resources to both people and animals. As a result, river-borne pathogens can cause widespread concern and can negatively affect society (Grill et al., 2019). However, our understanding of the fate of potential pathogenic bacteria at wastewater treatment plants (Li et al., 2015), urban recreational water (Cui et al., 2017; Fang et al., 2018) or groundwater (Chik et al., 2020) is much better compared to natural large-scale river ecosystems (Korajkic et al., 2019; Mathai et al., 2019).

As one of the longest rivers in Asia, the Yangtze River supports rich biodiversity and provides resources for over 40% people in China (She et al., 2019). Therefore, water-borne diseases caused by pathogenic bacteria in this region may create severe health risks to the general public. Particularly, the middle and lower reaches of the Yangtze River, including two vast adjoining lakes (Dongting Lake and Poyang Lake; thereafter as MLDP), covers one of the highly populated regions in China. Since land-based runoff pathogens from agricultural activities and urbanization (domestic sewage Cui et al., 2019, agricultural effluent Al-Gheethi et al., 2018 and livestock excreta Alegbeleye and Sant'Ana, 2020) are major sources of pathogens in the river, it is possible that these areas contain greater levels of bacterial pathogens than the upper reach of the Yangtze River. More importantly, the MLDP region is the sole habitat of the world's only freshwater porpoise, the Yangtze finless porpoise (*Neophocaena asiaeorientalis asiaeorientalis*), which has been listed as Critically Endangered with ~1,012 individuals left estimated in 2017 (Huang et al., 2019). Therefore, examining potential pathogenic bacteria in the MLDP region may be sufficient to reveal the bacterial pathogen distribution in the Yangtze River under anthropogenic activities, and can also provide basis for the habitat quality monitoring of the endemic Yangtze finless porpoise.

Microbial biogeography aims to reveal the spatial distribution patterns and their underlying factors, i.e., where they exist, what are they and at what abundance, and why (Martiny et al., 2006). Investigating the biogeographic patterns of bacterial pathogens in the river can help unveil the distribution of bacterial pathogens in space, and contribution of various environmental variables in structuring pathogen communities, which could aid in water managements by predicting pathogen responses to the global environmental changes. Studies on pathogens present in the Yangtze River mainly focused on their diversity and abundance in certain sections. Whereas, pathogen distribution patterns, based on a large-scale sampling and their underlying drivers, are limited (Sun et al., 2017).

In the present study, next-generation sequencing of the *16S rRNA* gene was applied to investigate the spatial dynamics and shaping factors of bacterial pathogens in the MLDP region. For this study we selected several environmental variables (i.e., water ions, heavy metals, water physicochemical properties, air temperature and land use types) and geographic distances as proxies. All of these factors have been shown to influence the biogeographic distribution patterns of microbial communities (Viau et al., 2011; Zhou et al., 2020). Here we primarily focused on the spatial variation pattern of the diversity and composition of bacterial pathogens in the Yangtze River and how different variables influence the pathogen community. This study provides detailed bacterial pathogen profiles, as well as their underlying drivers, all of which can provide a reference for a mechanistic understanding of the control of potential bacterial pathogens in the Yangtze River.

## Materials and methods

### Study site and sample collection

**Figure 1:**
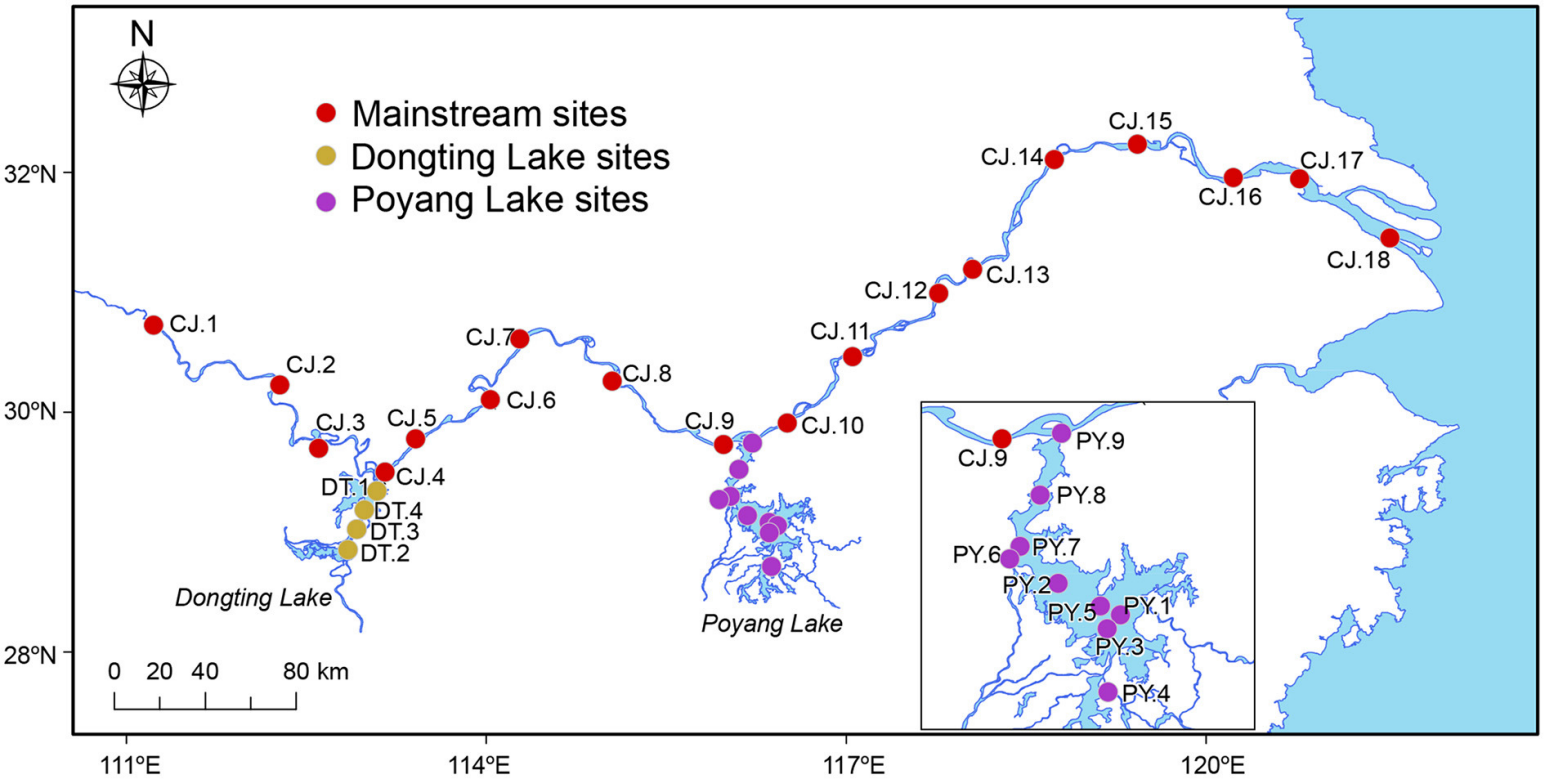
Sampling sites of this study. A total of 93 samples were collected at 31 sampling sites in the Yangtze River covering the middle and lower reaches of ~1,000 km, as well as its two major adjoining lakes (Dongting Lake and Poyang Lake). The 18 sites in the Yangtze River mainstream were divided geographically into three sections by Dongting Lake and Poyang Lake: MS1 (four sites: CJ.1–CJ.4), MS2 (five sites: CJ.5–CJ.9) and MS3 (nine sites: CJ.10–CJ.18). The map showed drainage during the dry season when our samples were collected (Nov–Dec 2017).

The third Yangtze Freshwater Dolphin Expedition was conducted between November and December in 2017. During this expedition, 93 water samples were collected from 31 sites, including 18 sites in the Yangtze River mainstream (middle and lower reaches, spanning a geographic distance of ~1,000 km), and 13 sites within the appended lakes, Dongting Lake (four sites) and Poyang Lake (nine sites; Figure 1). The 18 sampling sites along the Yangtze River mainstream were divided geographically into three sections by Dongting and Poyang Lake: MS1, MS2 and MS3 (Figure 1).

At each sampling site, triplicate water samples at 0.5 m below the surface were gathered as biological replicates using a VanDorn water container (KC-Denmark, Denmark) as described previously (Stewart et al., 2017). In brief, before each collection the container was first disinfected with 20% diluted bleach for 15 min, and then cleaned with sterile distilled water for three times. A total of three 1-L water samples per site were then passed through a mixed cellulose esters filter paper (47-mm diameter and 0.45-μm pore size) by applying a portable peristaltic pump (Toronto, Canada). The filters were stored with 95% ethanol until DNA extraction.

### Environmental and geographic variable measurements

Among all environmental variables, major water ions (F^−^, Cl^−^, SO${}_{4}^{2-}$, Na^+^, K^+^, Mg^2+^ and Ca^2+^, mg/L), heavy metals (Cu, Zn, As, Ni and Pb, μg/L) in water and physiochemical properties (total nitrogen/TN, total phosphorus/TP, total organic carbon/TOC, ammonium nitrogen/NH${}_{4}^{+}$-N, chemical oxygen demand/COD, nitrate nitrogen/NO${}_{3^{-}}$-N, total dissolved solids/TDS, chlorophyll-*a*/Chl-*a*, dissolved oxygen/DO, suspended solid/SS, soluble reactive phosphorus/SRP, mg/L) were determined in the laboratory using standard protocols (Hu et al., 2012; Ao et al., 2014). Other water physiochemical properties, including turbidity, water temperature (°C), water pH and electrical conductivity/EC (μS/cm) were determined *in situ* at a water depth of 50 cm with a YSI multiparameter probe (YSI-EXO1, OH, USA), and a Secchi dish was used to evaluate the water transparency (cm) (Ao et al., 2014). Air temperature (°C) was recorded by an air thermometer, and geographic variables (longitude and latitude of each site) were recorded using a portable GPS when water samples were collected. Six land use types, including agricultural land, forest land, grassland, built-up land (urban land), wetland and waters, were computed. Briefly, land use types of the buffer zone (10 km × 2 km) upstream of each sampling site were first extracted by applying ArcGIS 10.4 (Zhou et al., 2020). For each sampling site, the percentage of each land use type was then calculated using the Landsat 8 OLI remote sensing data.

### DNA extraction, sequencing and reads processing

The ZR Fecal DNA Kit (Zymo Research Incorporated, CA, USA) was applied to extract DNA from all water samples following standard protocols strictly. DNA quality was then assessed by a NanoDrop ND-1000 spectrophotometer (Thermo Fisher Scientific, Waltham, MA, USA).

The universal primers (341F-806R) were used to amplify the V3–V4 region of the *16S rRNA* gene (Roggenbuck et al., 2014). PCR products from each sample were pooled at equal molality and were then run on an Illumina MiSeq benchtop sequencer (Majorbio Company, Shanghai, China; 2 × 250 bp paired ends).

Raw sequencing reads were processed on the website pipeline (www.majorbio.com) to retain only high-quality sequencing data. Briefly, after removing singletons and chimeric sequences, operational taxonomic units (OTUs) were clustered by applying UPARSE (Edgar, 2013) at the cutoff of 0.97. The representative sequences of OTUs were then classified into specific taxonomic units using the Ribosomal Database Project (RDP) Classifier (Cole et al., 2014) based on the current SILVA database (release 132) using the 0.8 threshold (Quast et al., 2012). FastTree was used to construct the phylogenetic tree based on the representative sequences of OTUs (Castresana, 2000; Price et al., 2010). Each sample was randomly resampled to 25,688 sequences (the lowest number of sequences), which is ample to capture the bacterial diversity (Supplementary Figure 1).

### Pathogen prediction

To obtain the potential functions from *16S rRNA* gene sequences, the Functional Annotation of PROkaryotic TAXa (FAPROTAX) database was applied to predict the functional groups of water microbiome. The database covers more than 4,600 culturable bacterial species whose function profiles have been experimentally verified (Louca et al., 2016). Those OTUs belonging to no less than one pathogenic groups were regarded as potential pathogenic OTUs that may threaten humans, aquatic animals or plants.

### Statistical analysis

Alpha diversity indices (the number of observed OTUs and Faith's PD) were computed (R package {Picante}) (Kembel et al., 2010). Alpha diversity indices and the relative abundance of major pathogenic genre between groups were tested by applying Wilcoxon rank sum test. To assess the difference of microbial communities between groups, the non-parametric multivariate analysis of dissimilarity (Adonis) was applied, on the basis of Bray-Curtis and weighted UniFrac distances. Principal Co-ordinates Analysis (PCoA) was further calculated to visualize patterns of the community dissimilarity across samples.

To investigate the separate effects of environmental and geographic variables on microbial community composition, partial Mantel tests (Spearman correlation; R package {vegan}) were used (Martiny et al., 2006). To further explore the relative contribution of each variable in structuring bacterial communities, a two-step multiple regression on matrices (MRM) method was applied (Wang et al., 2017). Specifically, before applying the MRM, the collinear variables that were highly correlated (Spearman's ρ^2^ > 0.7) were discarded (R package {Hmisc}; i.e., water temperature, Ca^2+^, NH${}_{4}^{+}$-N, SO${}_{4}^{2-}$ and TDS; Supplementary Figure 2). On the basis of the result from the first MRM run, non-significant (*p* > 0.05) variables were removed, and all the other significant variables were rerun for the second MRM test. To determine the associations between pathogenic bacteria genera and all significant variables reported from the second MRM run, the Mantel test was further performed. We modeled the distance-decay relationship (DDR) by a linear regression between compositional similarities and geographic distance, based on taxonomic and phylogenetic beta diversity separately (Martiny et al., 2011) The fast expectation-maximization for microbial source tracking (FEAST) method (Shenhav et al., 2019) was used to assess the likely contributions of different sources to the pathogenic bacteria of the Yangtze River downstream imported from the upstream.

## Results

### Overall bacterial composition in water samples

We obtained 7,141 bacterial OTUs across 93 samples from 31 sampling sites. The water microbiome was dominated by the phyla Actinobacteriota (34.8%) and Proteobacteria (34.5%), followed by Bacteroidetes (12.7%) and Cyanobacteria (7.1%; Supplementary Figure 3). The dominant classifiable genera were *hgcI clade* (belonging to the order Actinomycetales, 13.5%), followed by *CL*500-29 *marine group* (belonging to the order Actinomycetales, 10.0%), *Limnohabitans* (belonging to the order Burkholderiales, 5.6%) and *Flavobacterium* (belonging to the order Flavobacteriales, 3.2%; Supplementary Figure 4).

### Potential pathogenic bacterial composition

**Figure 2:**
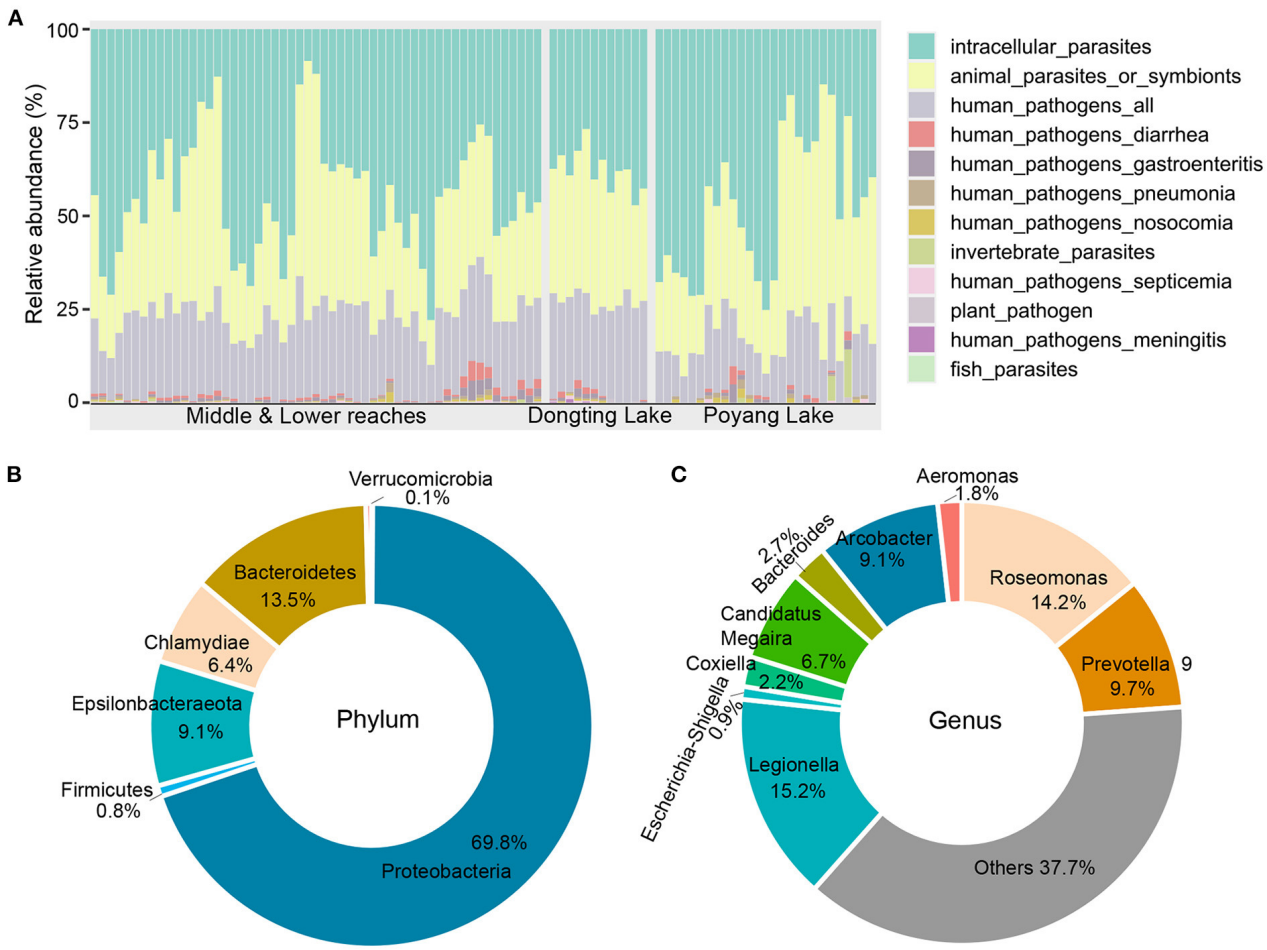
Functional and taxonomic composition of the potential pathogenic bacteria identified in this study. **(A)** Functional composition across all samples. **(B)** Taxonomic composition at the phylum level. **(C)** Taxonomic composition at the genus level.

A total of 375 OTUs (27,962 sequences) were assigned to 12 potential pathogenic groups. This accounts for an average of only 1.2% of the total abundance (Supplementary Figure 5A), suggesting that potential pathogenic bacteria present in the river tends to be low. The average relative abundance of potential pathogens was higher in samples collected from the mainstream (~3.1%) compared to the lakes (Poyang Lake, ~0.7%; Dongting Lake, ~1.7%; Supplementary Figure 5B). The 12 potentially pathogenic groups detected in this study included animal parasites or symbionts (AnPS), intracellular parasites (IntP), invertebrate parasites (InvP), human pathogens all (HuPA), human pathogens diarrhea (HuPD), human pathogens gastroenteritis (HuPG), human pathogens meningitis (HuPM), human pathogens nosocomia (HuPN), human pathogens pneumonia (HuPP), human pathogens septicemia (HuPS), plant pathogen (PlaP) and fish parasites (FisP; Figure 2A). Among the 12 potential pathogenic groups, intracellular parasites (IntP), animal parasites or symbionts (AnPS) and human pathogens all (HuPA) accounted for 97.5% of the total functional abundance (Figure 2A).

**Figure 3:**
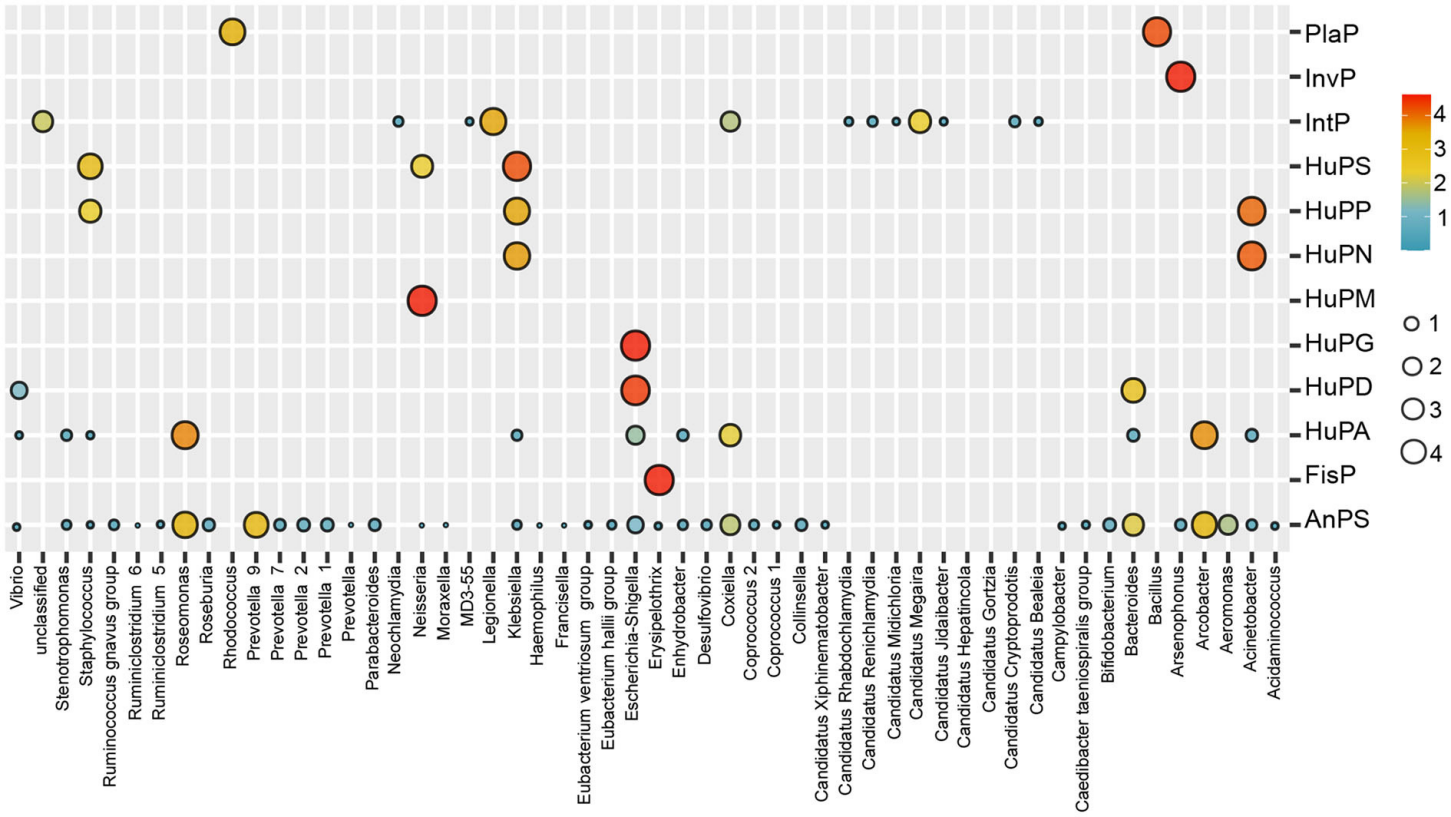
Bubble map showing the distribution of potential pathogenic groups in each pathogenic genus. AnPS, animal parasites or symbionts; IntP, intracellular parasites; InvP, invertebrate parasites; HuPA, human pathogens all; HuPD, human pathogens diarrhea; HuPG, human pathogens gastroenteritis; HuPM, human pathogens meningitis; HuPN, human pathogens nosocomia; HuPP, human pathogens pneumonia; HuPS, human pathogens septicemia; PlaP, plant pathogen; FisP, fish parasites.

The bacterial composition of potential pathogens was classified into seven phyla, 53 genera and 38 species. The seven phyla were Proteobacteria (69.8%), Bacteroidetes (13.5%), Epsilonbacteraeota (9.1%), Chlamydiae (6.4%), Firmicutes (0.8%), Actinobacteria (0.4%) and Verrucomicrobia (0.1%; Figure 2B). The top five abundant genera were *Legionella* (15.2%), *Roseomonas* (14.2%), *Prevotella* 9 (9.7%), *Arcobacter* (9.1%) and *Candidatus Megaira* (6.7%; Figure 2C). The distribution of potential pathogenic groups in each pathogenic genus are shown in Figure 3. Specifically, HuPN and HuPP were largely composed of members of the genera *Acinetobacter* (63.0 and 56.7%, respectively) and *Klebsiella* (37.0 and 33.3%, respectively). *Erysipelothrix* (100%), *Neisseria* (100%), *Escherichia-Shigella* (100%) and *Arsenophonus* (100%) were exclusively found in the pathogenic groups FisP, HuPM, HuPG and InvP, respectively. HuPA was mainly composed of members of the genera *Arcobacter* (40.9%) and *Roseomonas* (44.6%). HuPD contained mainly *Escherichia-Shigella* (82.3%) and *Bacteroides* (15.6%). HuPS contained mostly *Klebsiella* (69.8%) and *Staphylococcus* (20.9%). IntP contained mostly *Legionella* (33.9%) and no rank genera (43.9%). PlaP only contained *Bacillus* (70%) and *Rhodococcus* (30%; Figure 3).

### Spatial distribution patterns of the potential pathogen community

**Figure 4:**
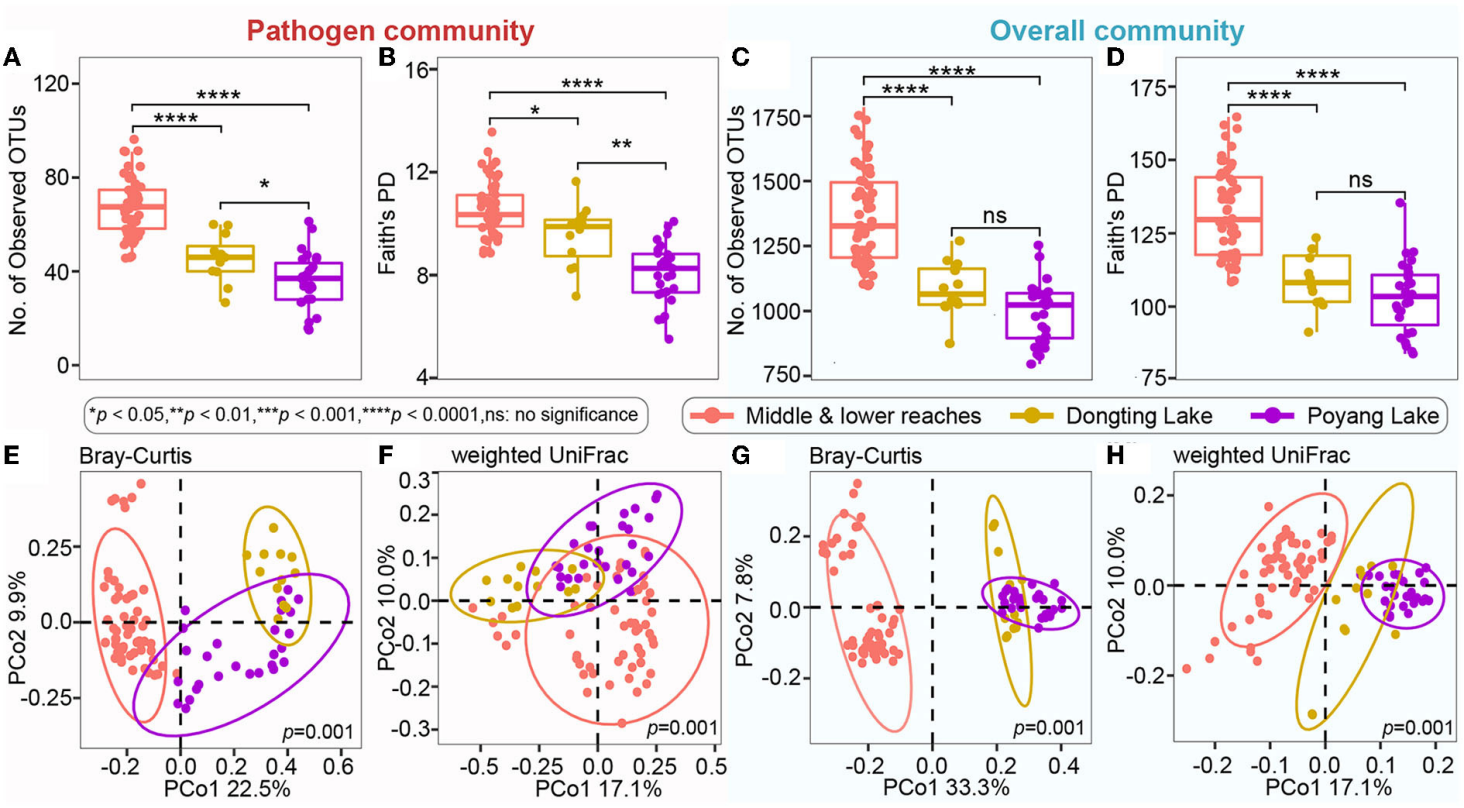
Microbial alpha diversity and composition of the pathogen and overall communities among different sections. **(A–D)** Number of observed OTUs and Faith's PD (phylogenetic diversity) indices in the pathogen community and overall community. The asterisk represents a significant difference between the two groups, *p < 0.05, **p < 0.01, ****p < 0.0001, ns: no significance. **(E–H)** Unconstrained PCoA with Bray-Curtis and weighted UniFrac distances that separated by different sections (*p* = 0.001, permutational multivariate analysis of variance by Adonis).

**Figure 5:**
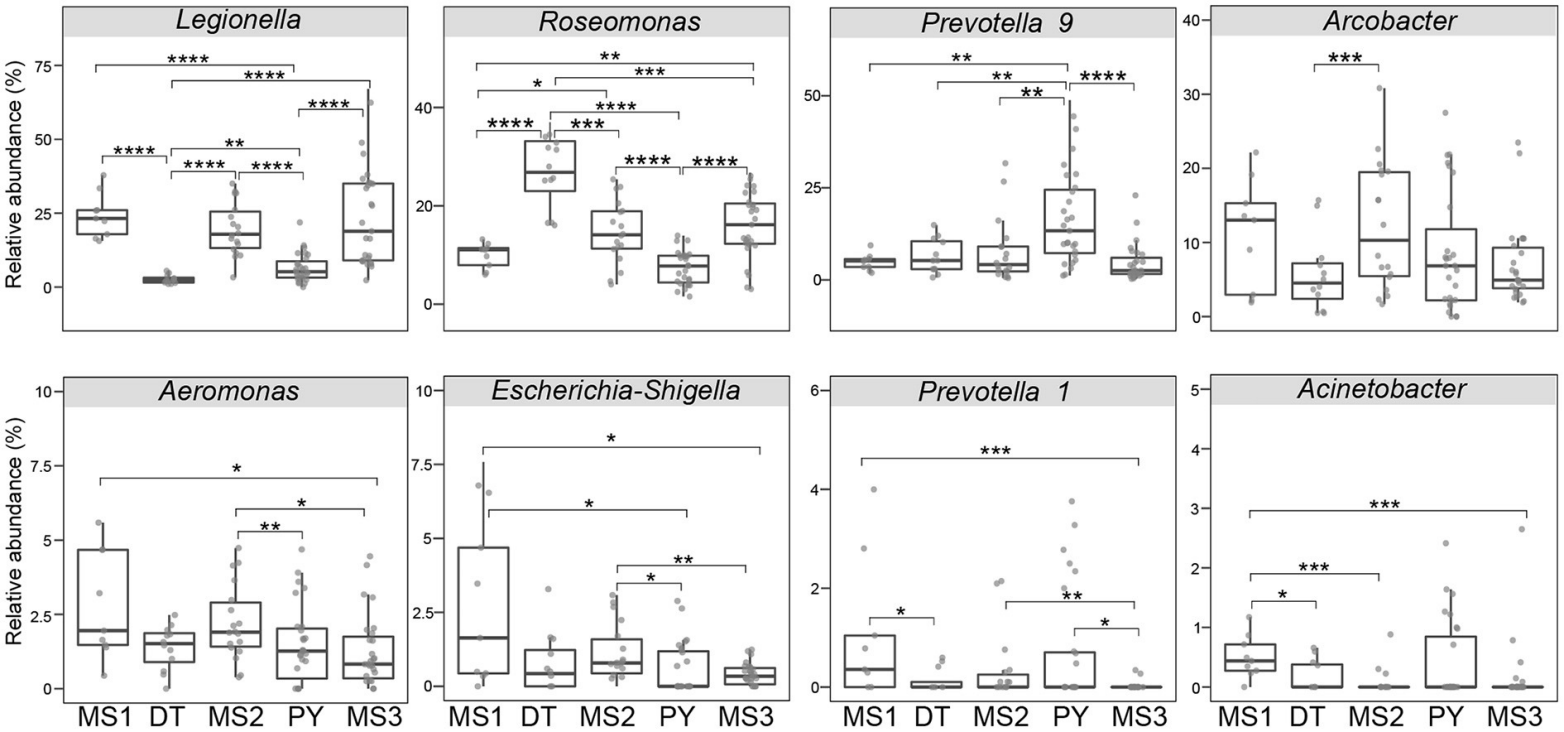
Relative abundance of major abundant pathogen genera in different sections. The asterisk represents a significant difference between the two groups, *p < 0.05, **p < 0.01, ***p < 0.001, ****p < 0.0001. DT, Dongting Lake; PY, Poyang Lake; MS, mainstream.

Even though microbial diversity indices of the overall bacterial community (covering both pathogen and non-pathogen communities) between Dongting Lake and Poyang Lake were comparable (Figures 4C,D), both the number of observed OTUs (Figure 4A) and phylogenetic diversity (Figure 4B) of the pathogen community from Dongting Lake were significantly higher than those from Poyang Lake. Additionally, the pathogen diversity increased significantly from the lakes to the mainstream (*p* < 0.05; Figures 4A,B). Among the mainstream samples, the pathogen diversity gradually decreased from MS1 and MS2 to MS3, with the MS2 section harboring a higher pathogen diversity (Supplementary Table 1). There were significant differences in the pathogen and overall community composition between the lakes and the mainstream, as visualized by PCoA plots based on the Bray-Curtis (Figures 4E,G) and weighted UniFrac distances (Figures 4F,H). Further statistical tests showed that the abundant pathogen genera exhibited significant spatial dynamics (Figure 5). Typically, *Legionella* was significantly enriched in the mainstream compared to the two lakes (Figure 5). However, *Roseomonas* and *Prevotella* nine were significantly enriched in Dongting and Poyang Lake, respectively (Figure 5).

Our results revealed that there were significant distance-decay relationships (DDRs) based on both taxonomic (Supplementary Figure 6A) and phylogenetic diversity (Supplementary Figure 6B) for the pathogen community along the Yangtze River, showing that the pathogen community became more dissimilar with increasing geographic distances. However, the slopes of DDRs varied between the pathogen community and the overall community (Supplementary Figure 6), indicating different driving forces shaping both communities. The FEAST analysis (Supplementary Figure 7) implied that 14.5%−42.6% of the pathogen community in the MS3 section were likely originated from the upstream MS1 (~17.3%), Dongting Lake (19.2%), MS2 (42.6%) and Poyang Lake (14.5%), with the relative proportion increasing from upstream to downstream.

### Linkages between the pathogen community and environmental and geographic variables

**Figure 6:**
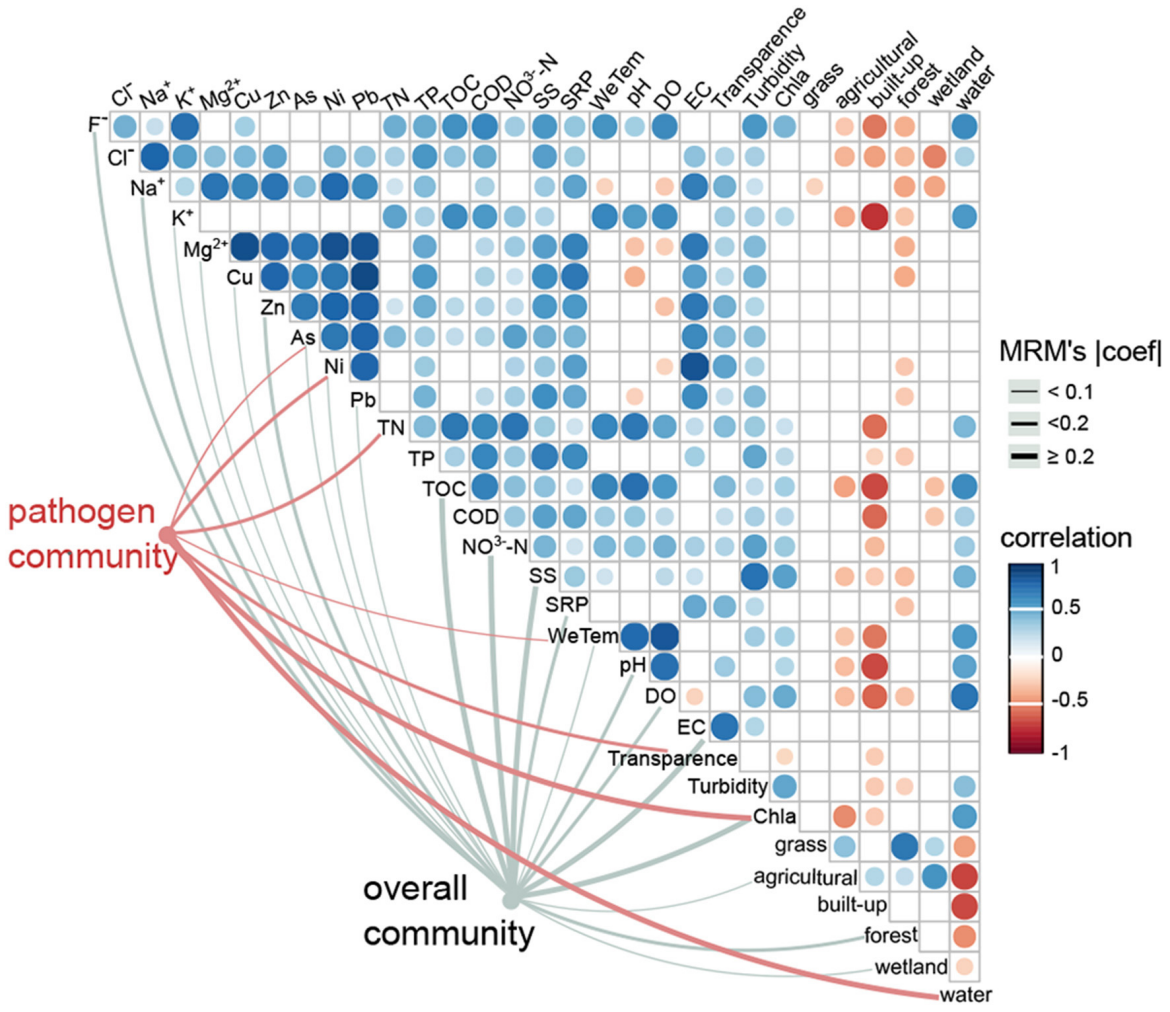
Effects of environmental drivers on the pathogen community and the overall community. The associations between environmental variables and the pathogen community (red edges) and the overall community (gray edges) were tested by MRM models (*p* < 0.05). The edge width corresponds to the MRM's coefficient. The color gradient indicates Spearman correlations among environmental variables (*p* < 0.05).

For the overall community, both environmental variables (*r* = 0.3, *p* < 0.001) and geographic distance (*r* = 0.1, *p* = 0.02) significantly affected the bacterial composition (Table 1). In contrast, the pathogen community was highly correlated with environmental variables (*r* = 0.5, *p* = 0.001), but was not correlated with geographic distances (*r* = 0.02, *p* = 0.37; Table 1). For the overall community, the MRM model explained up to 67.8% of the community structure (*p* < 0.001), with TOC (*b* = 0.6) and NO${}_{3}^{-}$-N (*b* = 0.4) being the most important variables (Figure 6, Supplementary Table 2). In contrast, only 35.3% of the pathogen community variability was explained (*p* < 0.001; Figure 6, Supplementary Table 2). In the pathogen community, Chl-*a* (*b* = 0.3), waters (*b* = 0.2), TN (*b* = −0.2), Ni (*b* = −0.1) and transparency (*b* = −0.1) contributed the larger partial regression coefficient, with other variables such as As and air temperature contributing a smaller but significant component (*b* = 0.04–0.05, *p* < 0.0; Figure 6, Supplementary Table 2).

**Table 1:** The effects of environmental variables and geographic distances on the pathogen community and the overall community based on the partial Mantel test.

**Explanatory matrices**	**Controlling for:**	**Pathogen community**		**Overall community**	
		***r***	***p*-Value**	***r***	***p*-Value**
Environmental variables	Geographic distances	**0.46**	**<0.001**	**0.32**	**<0.001**
Geographic distances	Environmental variables	0.02	0.345	**0.08**	**0.02**

Bold values indicate *p* < 0.05.

The linkage between major abundant pathogenic genre and the abovementioned seven significant environmental variables were examined by the Mantel test (Supplementary Table 3). The results showed that Chl-*a* and waters (land use type) were significantly correlated with the dominant genre *Legionella* (relative abundance, ~15.2%), *Roseomonas* (~14.2%), *Candidatus Megaira* (~6.7%) and *Coxiella* (~2.2%). Both TN and Ni also had significant correlations with *Roseomonas* and *Candidatus Megaira*. Air temperature was significantly linked with the most abundant pathogenic genre except for *Legionella, Aeromonas* and *Prevotella* 1 (~0.4%), whereas transparency was only significantly correlated with *Escherichia-Shigella* (~0.9%) and *Prevotella* 1. Notably, As was significantly correlated with *Roseomonas, Candidatus Megaira, Aeromonas* and *Prevotella* 1.

## Discussion

Given the contribution to public health issues from bacterial pathogens in aquatic ecosystems, an evaluation of bacterial pathogens may aid in preventing future human or animal infections. Here, by using sequencing-based profiling and taxonomy-based functional identification, we explored the distribution patterns of potential bacterial pathogens, as well as their driving forces in the middle and lower reaches of the Yangtze River, including two vast adjoining lakes (Dongting Lake and Poyang Lake), based on a large-scale sampling. A wide range of potential bacterial pathogens was detected, which could threaten human and animal hygienics in the Yangtze River. The bacterial community of potential pathogens exhibited significant biogeographic dynamics, with water physicochemical factors (i.e., Chlorophyll-*a*, transparency, total nitrogen), heavy metals (i.e., As and Ni), climate (air temperature) and land use type (i.e., waters) significantly structuring the potential pathogen community. Moreover, compared to the overall microbial community, a larger proportion (64.7%) of the pathogen community remained unexplained by the environmental and spatial processes we examined in this study.

Our results found many common and potentially pathogenic bacteria, including *Legionella* (frequency of occurrence 92/93 water samples, affiliated with the class Gammaproteobacteria and family Legionellaceae), *Roseomonas* (93/93 water samples, affiliated with the class Alphaproteobacteria and family Acetobacteraceae), *Prevotella* 9 (93/93 water samples, affiliated with the class Bacteroidetes and the family Prevotellaceae) and *Candidatus Megaira* (92/93 water samples, affiliated with the class Alphaproteobacteria and the family Rickettsiaceae) were prevalent across all 31 sampling sites. These potential pathogenic bacteria are capable of infecting humans, aquatic animals or plants. For example, *Legionella* spp. are aerobic Gram-negative bacilli which mainly survive in aqueous environments (Edelstein and Lück, 2015). These bacteria are intracellular pathogens of freshwater protozoa and utilize the same mechanism to invade human phagocytic cells, thus causing severe Legionnaires' disease (Fields et al., 2002; Kenagy et al., 2017). The genus *Roseomonas* has been frequently isolated from environment samples, such as drinking water, freshwater and marine invertebrates (Zhang et al., 2020; Li et al., 2021; Yin et al., 2021). Although the transmission route of *Roseomonas* infections is unclear, it's theorized that an infection could occur through contaminated water, and cause a disease, such as a bacteremia in immunocompromised individuals (Ioannou et al., 2020). In our study, *Arcobacter cryaerophilus* was detected at both a high relative abundance (9.1%) and frequency of occurrence (90/93 water samples). As one of the major zoonotic pathogenic *Arcobacter* species, *A. cryaerophilus* is associated with human gastrointestinal diseases (Collado and Figueras, 2011). Generally, the results of this study suggested that pathogenic genera were in low abundance (~1.2% average, Supplementary Figure 4B) and many other pathogenic bacteria were almost negligible, such as *Vibrio cholerae* (seven reads) and *Staphylococcus warneri* (nine reads). Therefore, focusing only on the abundant microbial taxa would thus overlook rare, but important pathogens. It should be noted that the dominant pathogenic genera in the Yangtze River differed greatly from those in other natural rivers, suggesting a river specificity of the pathogen community composition. For example, members of the genus *Arcobacter* were abundant in the Yangtze River (9.1%; Figure 2C), but were less (<1.8%) in the Pearl River (Zhou et al., 2020). *Acinetobacter* species were predominant in the Pearl River (35.0%) and extremely low in the Yangtze River (0.3%) (Zhou et al., 2020). Hence, strategies to effectively control certain pathogens in one river ecosystem might not be necessarily be applicable to others river ecosystems. The detected pathogens present in this study should in future be included in the water quality monitoring system of the Yangtze River and appended lakes to better prevent the spread of bacterial pathogens and disease outbreaks.

There are various possible origins of the water pathogens. Potential point sources (e.g., industrial wastewater and urban sewage) and non-point sources (e.g., land-based runoff containing wild and domestic animal excreta, leaking sewage, and agricultural effluent) have been identified as causes of pathogen contamination in natural river systems (Kim et al., 2005; Pachepsky et al., 2011). As both Dongting Lake and Poyang Lake flow into the Yangtze River mainstream, it's not surprising that the diversity and relative abundance of the pathogen community in the main stream were significantly higher compared to the lakes (Figure 4, Supplementary Figure S4). Interestingly, although the surface area of Dongting Lake is much smaller than that of Poyang Lake, significantly higher pathogen diversity was detected in Dongting Lake (Figures 4A,B). Since Dongting Lake receives relatively higher domestic sewage and industrial effluent and Poyang Lake receives more agricultural wastewater discharges (Li et al., 2013; Yuan et al., 2019), it's likely that, compared with Poyang Lake, Dongting Lake receives more diverse pathogens from domestic sewage and industrial effluent and less water exchange with the Yangtze mainstream, especially in the dry season, which may finally result in a ‘pathogen sink' of Dongting Lake. Evidently, the pathogen diversity decreased from upstream (MS1 and MS2) to downstream (MS3; Supplementary Table 1). This was in contrast with previous findings in the Pearl River, where the pathogen diversity increased downstream (Zhou et al., 2020). We speculated that, even though the Yangtze River downstream received pathogens from the upper stream, the inflow from other tributaries and surface runoffs, including extreme rainfall events, may dilute pathogen concentrations in the downstream. Therefore, a long-term monitoring is required to better understand the spatio-temporal distribution patterns of pathogens in the Yangtze River.

A good understanding of the factors that govern water pathogens is essential for improving predictions of water pathogen dynamics, and their responses to environmental change. Instead of only considering water physicochemical factors, this present study enhanced our understanding of the associations between the pathogen community and other multiple variables. Here the pathogen community composition was significantly correlated with water physicochemical factors (i.e., Chlorophyll-*a*, transparency, total nitrogen), heavy metals in the river (Ni and As), climate (air temperature) and land use type (i.e., waters). It has been shown that there is a direct correlation between the total number of bacterial cells and with the concentration of Chlorophyll-*a* (i.e., algal biomass) (Apple et al., 2008). The significant effect of Chlorophyll-*a* on the pathogen community, as demonstrated in the present study as well as in the previous study (Zhou et al., 2020), implies that the pathogen community could be regulated *via* controlling the phytoplankton amount. The effects of water transparency on the pathogen community could be due to the bactericidal effects of the solar radiation and its ability to cause direct DNA damage to bacteria, such as the fecal indicators *Escherichia coli* and *Pseudomonas aeruginosa* (Berruti et al., 2021). High turbidity substantially reduced the elimination efficiency of pathogens. Therefore, water transparency may aid in the inactivation of certain pathogens. The correlation between total nitrogen and heavy metals and the pathogen community may reflect detrimental human activities along coastal regions. A high-nutrition load and heavy metal-contaminated of wastewater are important sources of nutrients for water pathogens (Jin et al., 2018; Yang et al., 2020). In this study, air temperature on the sampling date, rather than water temperature as demonstrated previously (Zhou et al., 2020), was significantly associated with the pathogen community (Figure 5). Temperature has been considered to be one of the most important abiotic factors controlling microbial communities by regulating cell growth and reproduction (Zhou et al., 2016; Sun et al., 2018). The lack of water temperature correlation may because that the variation of the water temperature among sampling sites was less compared to air temperature. Or perhaps all of the water samples collected were within an optimal temperature range for the growth of potential pathogens. The significant effect of land use type (i.e., waters) on the pathogen community implied that anthropogenic activities-induced land use changes, may act as important sources of water pathogens. Even though many other processes, such as stochastic events may help explain the larger unexplained proportion (64.7%) of the pathogen community in this study, these environmental variables could contribute to predicting the bacterial pathogenic responses of aquatic ecosystems to anthropogenic pressures and global environmental change. Additionally, as different pathogenic genre exhibited different responses to environmental variables, and/or the environmental variables selectively enriched a given pathogenic genre, as shown in this study (Supplementary Table 3), focusing on individual pathogenic taxa may improve the prediction and management of water pathogens.

It should be noted that the sampling in the present research was conducted during one field survey in the dry season. Additional work, including sampling in tributaries, investigating the influences of rainfall and storm events, and seasonal changes on pathogen loads are still necessary to fully understand pathogen sources and dynamics along the Yangtze River. In this study, statistical associations between the pathogen community and environmental variables were observed. However, laboratory experiments are still required to establish the evidence base for possible causal links between pathogens and their influencing factors. It is also important to notice that functional annotations relying on the database FAPROTAX only provide inferred functions based on the *16S rRNA* fragments, that may not be as accurate as a genuine shotgun metagenomic study, which should be taken into account when considering the precision of pathogen assignment in this study.

In summary, by analyzing the bacterial communities presented in the middle and lower reaches of the Yangtze River, a wide-ranging pathogenic bacteria was detected that could menace public health. In this study, biogeographic dynamics of these potential pathogenic bacteria and their underlying drivers were also explored. Our research highlights the importance of preventing potential bacterial pathogens in the Yangtze River.

## Data availability statement

The datasets presented in this study can be found in online repositories. The names of the repository/repositories and accession number(s) can be found at: https://www.ncbi.nlm.nih.gov/, PRJNA837026.

## Author contributions

JZ designed this research. JL collected the samples and performed the lab experiments. XW, SW, and JL completed data analysis. XW, SW, and JZ wrote the manuscript. XW, FF, RM, KW, and DW assisted with experiments and advice on manuscript. All authors contributed to discussions and revisions, and approved the final manuscript.

## Funding

This study was partly supported by grants from the National Natural Science Foundation of China (No. 31870372), the Bureau of Science & Technology for Development, Chinese Academy of Sciences (No. ZSSD-004), and China Postdoctoral Science Foundation (No. 2020M682530).

## Conflict of interest

Author SW was employed by Changjiang Survey, Planning, Design and Research Co., Ltd.

The remaining authors declare that the research was conducted in the absence of any commercial or financial relationships that could be construed as a potential conflict of interest.

## Publisher's note

All claims expressed in this article are solely those of the authors and do not necessarily represent those of their affiliated organizations, or those of the publisher, the editors and the reviewers. Any product that may be evaluated in this article, or claim that may be made by its manufacturer, is not guaranteed or endorsed by the publisher.
