# Supplementary material for: Biogeographic patterns of potential pathogenic bacteria in the middle and lower reaches of the Yangtze River as well as its two adjoining lakes, China
Source: Front Microbiol. 2022 Sep 2;13:972243. doi: 10.3389/fmicb.2022.972243 (PMC9479215; doi:10.3389/fmicb.2022.972243)
Supplement: Supplementary file 1 [file Data_Sheet_1.docx]

Supplementary Material


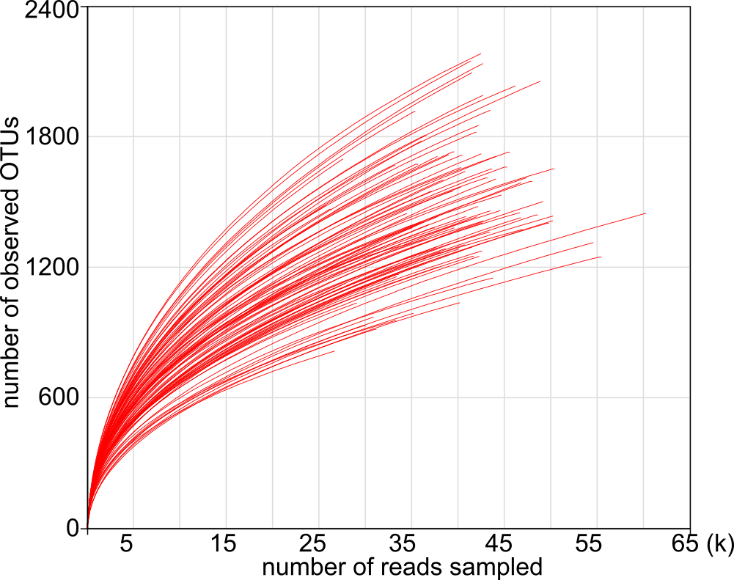


**Supplementary Figure 1.** The rarefaction curves of all samples in the study.


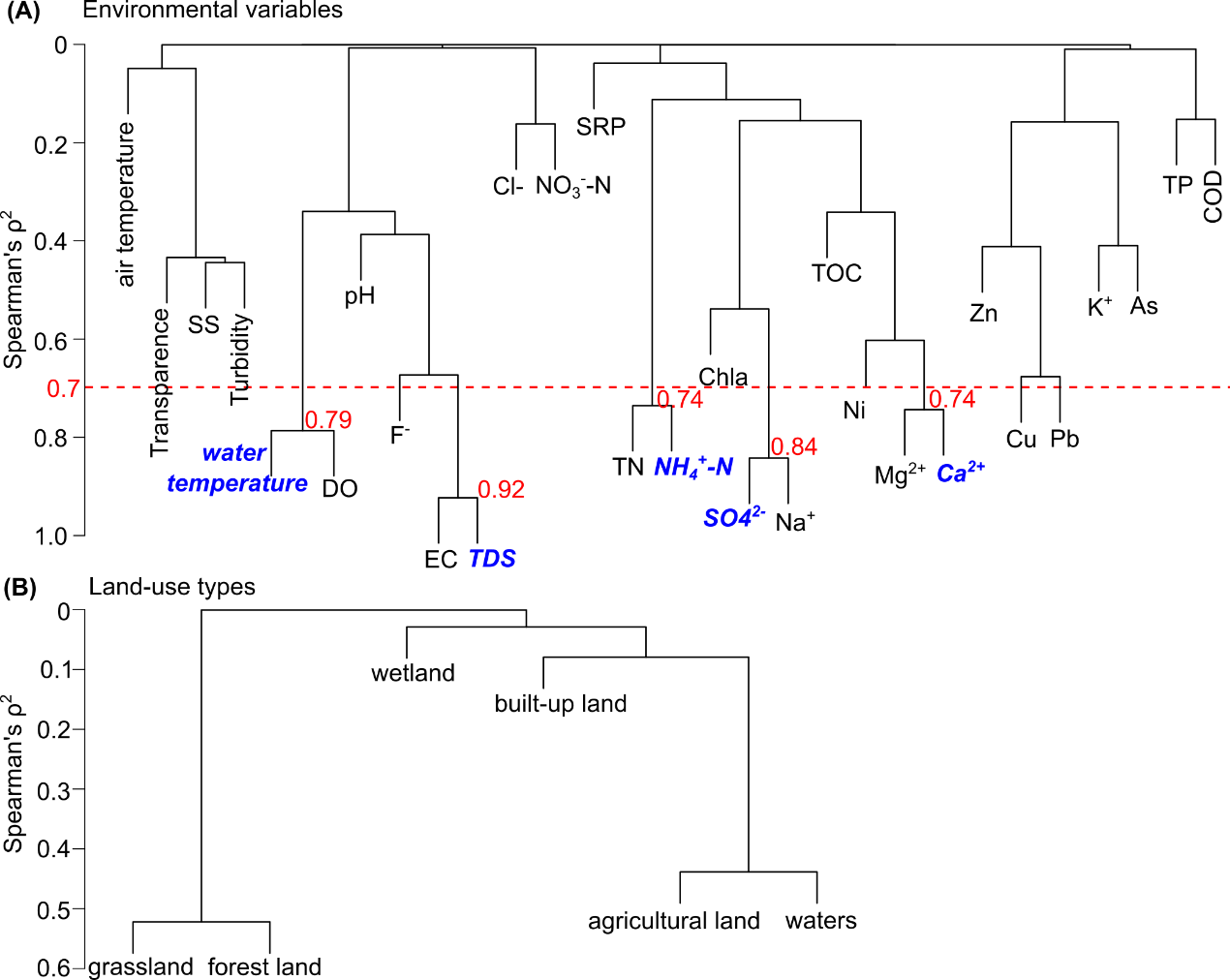


**Supplementary Figure 2.** Cluster analysis of the measured environmental variables. A, water physiochemical, major water ions, heavy metals and air temperature variables. B, land use types variables. Variables highlighted in blue with a higher correlation (Spearman’s *ρ*^2^ > 0.7) were removed before applying MRM tests.


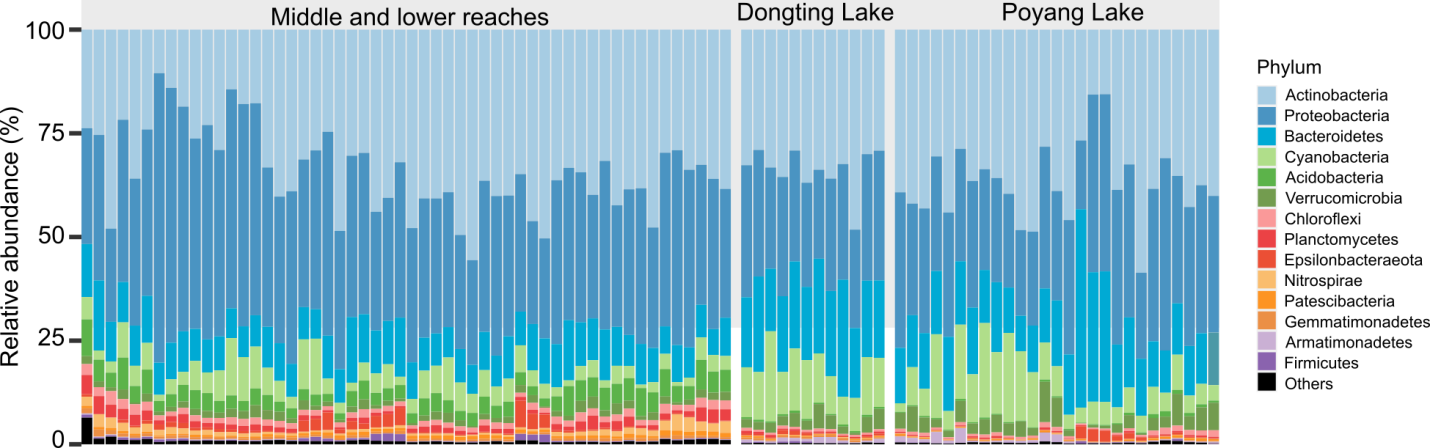


**Supplementary Figure 3.** Taxonomic composition of the overall microbial community across all samples at the phylum level.


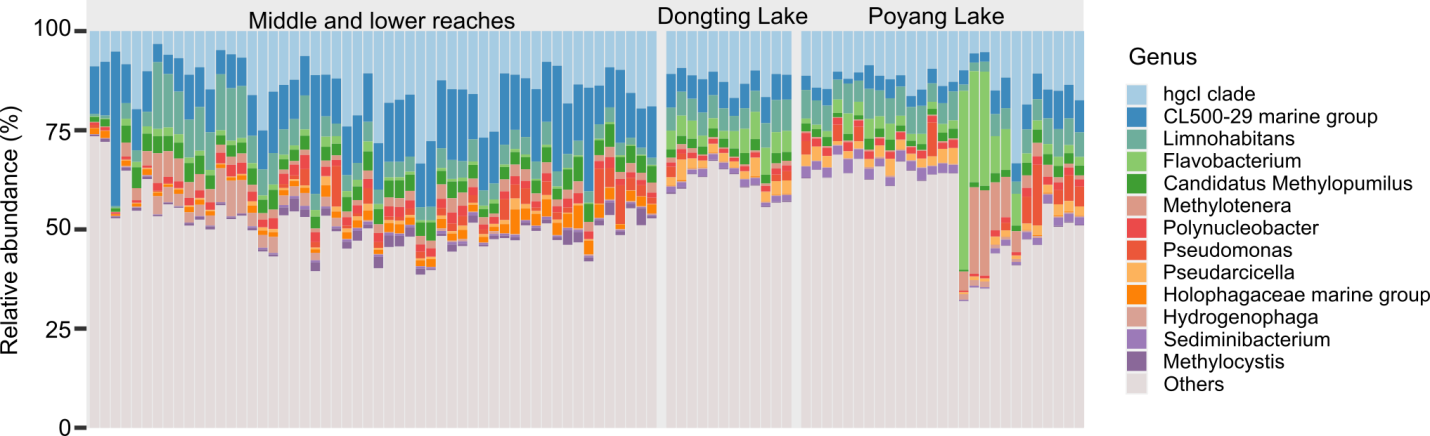


**Supplementary Figure 4.** Taxonomic composition of the overall microbial community across all samples at the genus level.


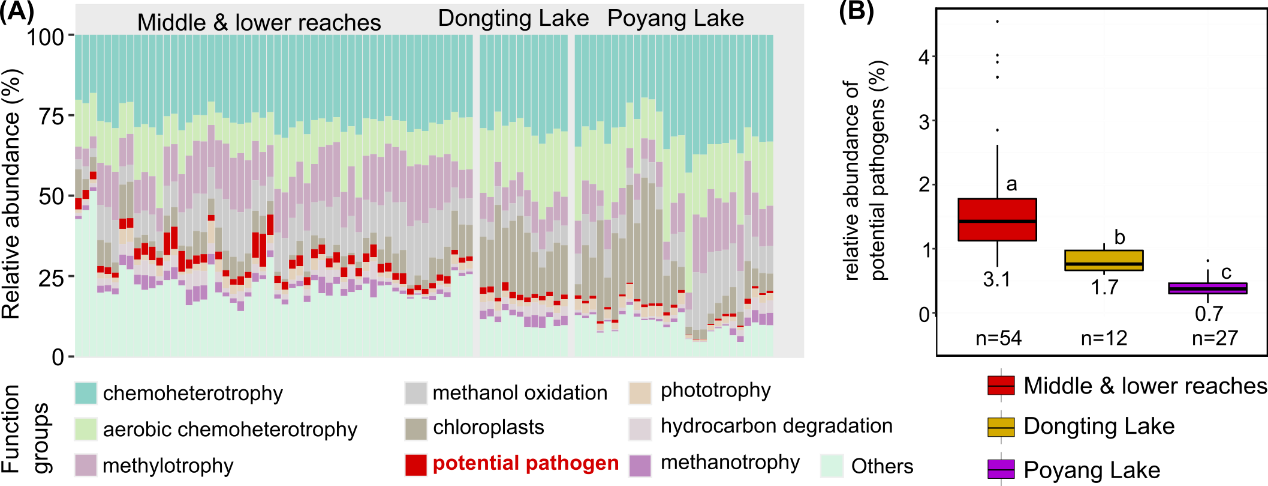


**Supplementary Figure 5.** Relative abundance of predicted functional groups and pathogens across all samples. A, stacked bar plot of the relative abundance of 10 major functional groups. B, relative abundance of potential pathogens among different sections along the Yangtze River.


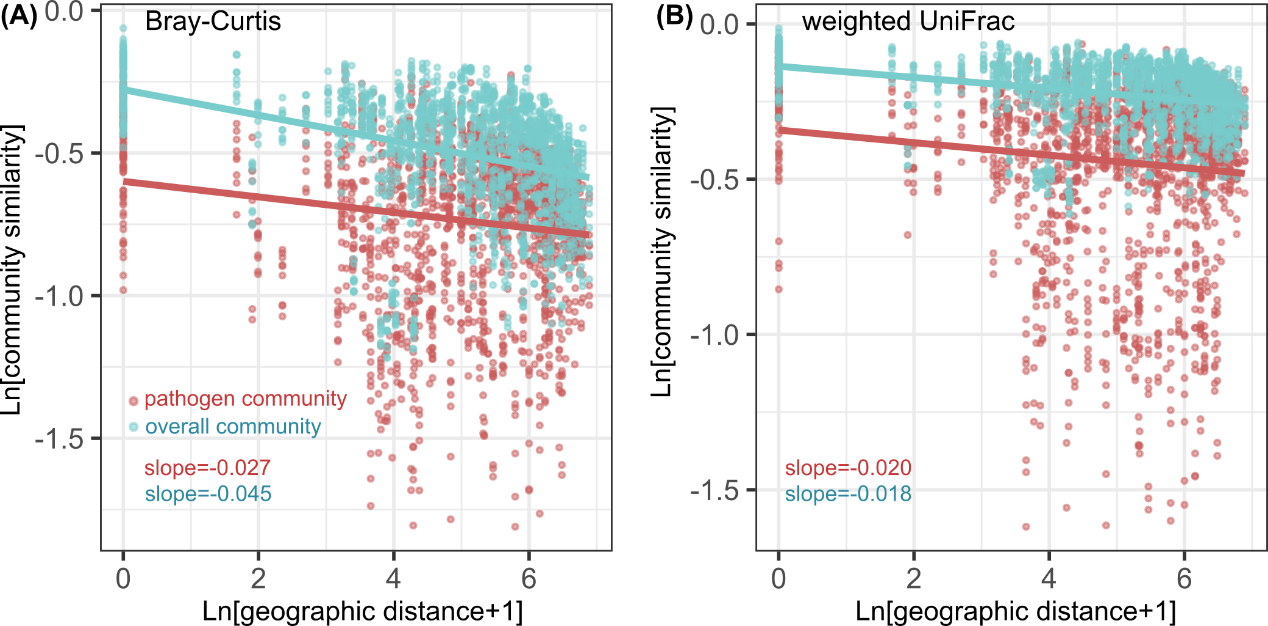


**Supplementary Figure 6.** The distance-decay curves of microbial similarities for both the pathogen community and the overall community (OTUs defined at the 97% sequence similarity). A, taxonomic diversity measured by weighted Bray-Curtis. B, phylogenetic diversity measured by weighted UniFrac.


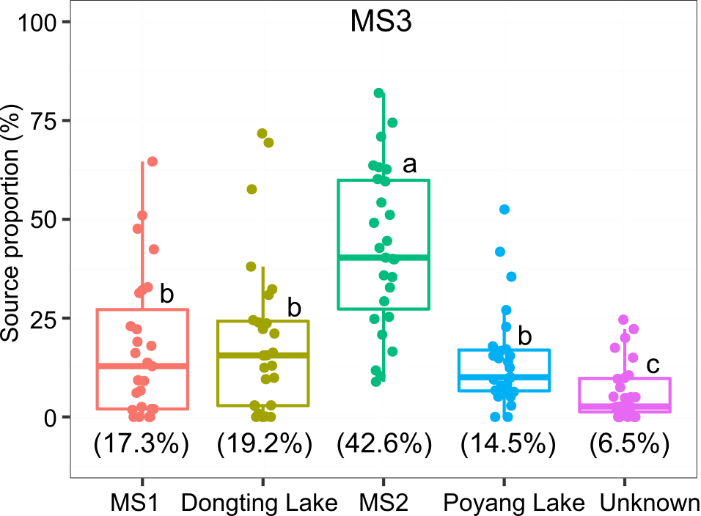


**Supplementary Figure 7.** Estimation of potential pathogen community in the downstream (MS3 section) that came from the upper stream. Bracketed text indicates the mean value of the source contribution from each group.

**Supplementary Table 1.** Alpha diversity indices (mean ± SD, Standard Deviation) of the pathogen community among different sections in the mainstream (MS).

| Group | No. of observed OTUs | Faith’s PD |
| --- | --- | --- |
| MS1 | 65.9 ± 9.8^a^ | 10.2 ± 0.9^a^ |
| MS2 | 72.7 ± 13.6^a^ | 10.5 ± 1.3^a^ |
| MS3 | 64.7 ± 11.0^a^ | 9.5 ± 1.0^b^ |

Note: Means superscripted by different lowercase letters in the same column are significantly different at the *p* < 0.05 level based on Wilcoxon rank sum tests.

**Supplementary Table 2.** Results of MRM analyses for the pathogen community and the overall community.

|  |  | Pathogen community  R^2^=0.353*** | overall community  R^2^=0.678*** |
| --- | --- | --- | --- |
| Geographic distances |  | -0.082*** | -0.108*** |
| Environmental variables | F^-^ | ND | -0.080** |
|  | Na^+^ | ND | -0.173* |
|  | K^+^ | ND | -0.090*** |
|  | Mg^2+^ | NS | 0.057* |
|  | Cu | ND | 0.071* |
|  | Zn | ND | 0.120*** |
|  | As | 0.053* | -0.057* |
|  | Ni | -0.120*** | 0.070* |
|  | Pb | NS | -0.069*** |
|  | TN | -0.163*** | NS |
|  | TP | NS | NS |
|  | TOC | NS | 0.478*** |
|  | NO_3_^-^-N | ND | 0.372*** |
|  | SS | ND | 0.327*** |
|  | SRP | ND | -0.129*** |
|  | air temperature | 0.041* | -0.026* |
|  | pH | ND | 0.120*** |
|  | EC | NS | -0.253* |
|  | Transparency | -0.117*** | NS |
|  | Chl-*a* | 0.268*** | -0.312*** |
|  | Agricultural land | ND | 0.055*** |
|  | Waters | 0.176* | NS |
|  | Built-up land | NS | NS |
|  | Forest land | ND | 0.108*** |

Note: ND, not determined (removed by the varclus results); NS, not significant. * *p* < 0.05, ** *p* < 0.01, *** *p* < 0.001. Abbreviations: TN, total nitrogen; TP, total phosphorus; TOC, total organic carbon; NO_3_^-^-N, nitrate nitrogen; SS, suspended solid; SRP, soluble reactive phosphorus; EC, electrical conductivity; Chl-*a*, chlorophyll-*a*.

**Supplementary Table 3.** Correlations between major abundant pathogenic genre and significant environmental variables using Mantel tests.

| Genus | Relative abundance | Chl-*a* | Waters | TN | Ni | Transparency | As | Air temperature |
| --- | --- | --- | --- | --- | --- | --- | --- | --- |
| *Legionella* | 15.2% | 0.229 | 0.098 |  |  |  |  |  |
| *Roseomonas* | 14.2% | 0.272 | 0.374 | 0.071 | 0.491 |  | 0.389 | 0.141 |
| *Candidatus Megaira* | 6.7% | 0.171 | 0.111 | 0.212 | 0.253 |  | 0.160 | 0.120 |
| *Bacteroides* | 2.7% |  |  |  |  |  |  | 0.183 |
| *Coxiella* | 2.2% | 0.259 | 0.091 | 0.229 |  |  |  | 0.375 |
| *Aeromonas* | 1.8% |  |  |  |  |  | 0.126 |  |
| *Escherichia-Shigella* | 0.9% |  |  |  |  | 0.281 |  | 0.229 |
| *Prevotella1* | 0.4% |  |  |  |  | 0.242 | 0.153 |  |

Only Mantel’s r values with *p* < 0.05 were showed in the table. Abbreviations: Chl-*a*, chlorophyll-*a*; TN, total nitrogen.
